# Retraction: Gliske, A New Theory of Gender Dysphoria Incorporating the Distress, Social Behavioral, and Body-Ownership Networks

**DOI:** 10.1523/ENEURO.0149-20.2020

**Published:** 2020-04-24

Retraction for “A New Theory of Gender Dysphoria Incorporating the Distress, Social Behavioral, and Body-Ownership Networks” by Stephen V. Gliske which published online on December 2, 2019.

Based on concerns about this article’s validity, the *eNeuro* Editorial Board commissioned an in-depth postpublication review of this article by qualified experts in the field and senior members of the Editorial Board. Their consensus is to retract the article “because of major flaws, including circular reasoning, the lack of supporting evidence in the literature, a noncritical use of the available literature, and confusion in terminology. […] The links drawn between at least two of the three networks are not compelling based on the small base of literature cited, especially since this literature is only of structural but not functional nature. Many of the present statements regarding functional implications are sheer conjecture. There is not enough evidence justifying this ‘new’ theory and how it would actually advance the field.” The full rereview synthesis statement is available online as Extended Data.

10.1523/ENEURO.0149-20.2020.ed1 Extended Data Synthesis statement on “A New Theory of Gender Dysphoria Incorporating the Distress, Social Behavioral, and Body-Ownership Networks”. Download Extended Data, PDF file.

Based on this recommendation, the editors are retracting this article.

