# Supplementary material for: Retraction: Gliske, A New Theory of Gender Dysphoria Incorporating the Distress, Social Behavioral, and Body-Ownership Networks
Source: eNeuro. 2020 Apr 24;7(2):ENEURO.0149-20.2020. doi: 10.1523/ENEURO.0149-20.2020 (PMC7294453; doi:10.1523/ENEURO.0149-20.2020)
Supplement: Extended Data — Synthesis statement on “A New Theory of Gender Dysphoria Incorporating the Distress, Social Behavioral, and Body-Ownership Networks”. Download Extended Data, PDF file. [file enu-eN-RET-0149-20-s01.pdf]

Synthesis statement on “A New Theory of Gender Dysphoria Incorporating the Distress, Social Behavioral, and Body-Ownership Networks”

Reviewing Editor: Julie Bakker, University of Liege

Contributing Editors: Rae Silver, Margaret McCarthy, and Christophe Bernard

Decisions are customarily a result of the Reviewing Editor and the peer reviewers coming together and discussing their recommendations until a consensus is reached. When revisions are invited, a fact-based synthesis statement explaining their decision will be listed below. The following reviewers agreed to reveal their identity: Ute Habel, Sven Muller, and Antonio Guillamon Fernandez.

Three reviewers and I have re-reviewed your paper submitted to eNeuro in the New Theory section. The consensus was to retract the paper because of major flaws including circular reasoning, the lack of supporting evidence in the literature, a non-critical use of the available literature, and confusion in terminology. Details are provided in the following synthesis of our discussions.

Flaws regarding the construction of the theory

A theory is proposed that chronic distress, gender nonconformity and incongruence, and body ownership networks would be related. In order to build the theory, the following logic is used:

1. The author hypothesizes that chronic distress, gender nonconformity and incongruence, and body ownership networks would be related.
2. The author looks *for information* in the literature about the implication of brain regions and networks in relation to the hypothesis to obtain evidence.
3. Thinking to have obtained evidence, the author confirms the hypothesis and then jumps to propose a theory.

This is not the formal way to build up a theory or to verify hypotheses. According to Hempel, hypotheses are invented from observations and then verified by experimental designs. The way followed by the author is somewhat circular: He or she proposes a hypothesis that he/she has observed in the literature and then confirms it in the literature. Following the sort of thinking used, why not include networks related to the autism spectrum since transgender people score high in some questionnaires? One could add networks to the theory *ad libitum*.

When dissecting the theory: 3 different networks have been proposed to be involved: (1) a social behavioral network, (2) a distress anxiety and fear network and (3) a body ownership network. At

present, there are very few experimental data available supporting a role for the first 2 networks, and in particular no functional data. Even if one accepts this way to build up theories, the management of the literature by the author does not help very much because the author uncritically accepts all the bibliography employed to the write the essay.

Regarding the section “Dimension 1: chronic distress”: the author affirms at the beginning “The key neuronal substrate for processing distress is the central extended amygdala, which includes the BNST and central amygdala”. The following literature used to support this idea:

1. Lebow an Chen (2016): A general review of the BNST.
2. Newmann (1999): A general review on the extended amygdala mainly in rodents, includes BNST
3. O’Connell & Hofmann (2011): phylogenetic review of the mesolimbic reward system, includes BNST
4. Zhu et al (2014): rhythm of expression of PER2, includes BNST.
5. Saper et al (2005a and b): regulation of sleep, emotions, does not include BNST.
6. Zhou et al (1995): BNSTc feminized in transwomen.
7. Tillman et al (2018): MRI functional connectivity of BNST with central amygdala.
8. Zubiaurre-Elorza et al (2013): structural MRI comparing trans men, trans women, cis men and women: larger cortical volume of trans men, trans women and cis women than cis men. It is interpreted as altered in transgender.
9. Manzouri et al (2017): structural and functional MRI in trans men and the cis control. mid-frontal, precuneal-parietal, and lingual cortex than both male and female controls and weaker connectivity in frontoparietal regions.

Problems in dealing with this literature:

- Refs. 1, 2, 3, 4 and 5 are taken as a whole. The author should detect references that are directly relevant for the hypothesis.
- Ref 6 is the classic work by the Swaab lab. It is accepted without criticism that it is based on postmortem specimens of hormonally treated trans women. In fact, the unique postmortem work that cannot be criticized for being hormonally treated is the work on the infundibular nucleus because estrogens treatment predicts a decrease in volume (Zubiaurre-Elorza et al, 2014) while *post mortem* specimens of trans women showed a feminine pattern (Taziaux et al, 2012, 2016).
- In ref. 8 the author misses the developmental explanation by the work’s authors.
- In ref 9 the author misses the relationships and differences with ref 8.

All these details suggest that the author does not know (or avoids) the intricacies and complexities of the literature on transgender people and the explanation for the lack of coincidence between research groups.

More specifically, much is based on the study from the group of Swaab, which is seminal. However, the data are a lot less clear than they are made to sound in the paper and thus the

theory is on shaky foundations since that finding appears essential for the theory. The BNST finding in trans persons was in a very small sample (<10) of post-mortem brains and most of the individuals were using hormone treatment. Although Swaab and colleagues tried to account for this, it is statistically unsound and methodologically impossibly (actually) simply because they (of course) had no pre-treatment brain samples. Thus, all they had was a tiny sample of post-mortem brains that had been treated with hormones for varying number of years. Thus, a lot of the "logical" jumps from existing structural findings to proposed functional contributions that appear throughout the text are non-sequiturs. In addition, the BNST do not cover all the (chronic) distress networks, in which for example especially amygdala and hippocampus are heavily involved as well as other networks, such as the salience network (see vanOort et al., 2017). The Tillmann study does not refer to a distress network, and the regions mentioned are not only involved in distress processing. So, the justification for this first element of the theory is not strong.

It is also particularly striking that the author has not made use of the only available line of enquiry that does happen to have structural and functional data, namely olfaction. Why are these data not discussed at all? In light of the lack of fMRI studies in the field in general and networks 1 and 2 in particular, olfaction is one of the few areas where researchers have converging structural, functional, and behavioral data all pointing into the same direction. All conclusions about the other (or new) networks are based on structural data that lack any functional and/or behavioral equivalents. Why has this line been ignored in the theory?

Regarding the fear network, there are no functional data available in transgender people at present. The third network is the one that has the most neuroimaging data, but also regarding these data there is an oversimplification of interpretation as well as a selection made of what could be used to build this theory. More specifically, in figure 1, the network(s) with the least neuroimaging evidence available in trans people (especially network 1) portrays all the possible connections between brain regions. These connections, of course, are all based on either structural data or data from outside the trans population but with no real evidence of functional activations. Likewise, presently there is no neuroimaging paper on the fear and anxiety network in trans people again leaving the reader wondering how the conclusions are drawn in the absence of empirical data. By contrast, the network (3) with the most neuroimaging data available (mostly Savic et al.), strikingly shows no arrows or connections drawn between any of the brain regions. Thus, there is a clear mismatch between the available evidence, the proposed detail of knowledge in the different networks, and the conclusions drawn from it by the authors. A related question is thus: what role is played by all the other brain imaging findings that occurred in the cited studies but that did seem to be relevant for the current theory. In particular, the social behavior network refers to many functions, as outlined, so the link to gender nonconformity is not clear and there is no convincing evidence or reference, just mentions that some of the regions of the network are altered in transgender. Since the author explicitly states that the theory refers to gender dysphoria (and not transgender – see below), this evidence shouldn't even be used. The overall picture thus seems to be incomplete and selective. Of note, in any case, much of the structural findings are, as the author rightly notes, based on small samples and larger replication efforts are

needed thus casting a big shadow of doubt on the stability and reliability of the findings in the first place.

Finally, there is a major issue regarding the central statement of causality of “being trans.” The author notes that current work is correlational and does not include the functional aspects (of the different networks) sufficiently. Instead, they describe that a functional interaction (to varying degrees) between the 3 networks is “causing” the “transgender experience.” Given that the author is basing their new theory, for the most part, on brain structural data, it could be asked how they are avoiding this criticism of being correlational. Thus, there is a new theory of gender identity being proposed that is concocted by throwing in various studies, all correlational in nature, to evoke a new theory that is causational. This constitutes a major weakness.

<u>Confusing terminology, including mixing gender dysphoria and transgender issues</u>

In terms of language, the paper is in many parts not following adequate terminology (please see the EPATH/WPATH guidelines on language when describing trans people). MtF/FtM, transsexual etc., which appear amply in the text are no longer adequate terms to be used and should be adjusted for a more respectful language. Similarly, the author appears disrespectful to the community because he/she does not seem to believe that a transgender person’s statement of belonging to one or the other gender is taken at face value and believed to be true. For example, saying that I did not believe a trans woman’s statement that she is a woman is as equally ludicrous as saying that I did not believe that a person saying that they are sexually attracted to a person from the same sex. On this note, highly annoying while reading the paper is the impression the reader gets by the overuse of the first person singular “I”, which, within the context of the various descriptions of trans people appears highly condescending.

Certain sections raise some issues (cf.“additional relevant data”). Because these sections are not well motivated and/or integrated they appear haphazard and out of place. For example, what exactly is the author trying to say in the paragraph noting that trans people have been found to have elevated presence of autistic traits within relation to their theory? Of note, in that section it is said, “but outcome measures directly related to distress or body ownership have not typically been considered or reported in the past”. There are several reviews including 77 studies to effects of surgery and hormonal therapy (Defreyne et al., 2017). Life quality has been assessed, satisfaction, and showed improvement. Puberty suppression also showed high satisfaction (de Vries et al., 2014). In 67 transgender people, of whom 73% received hormonal therapy, this led to higher self-esteem, less depression, and a better psychological life quality (Gorin-Lazard et al., 2013). In a longitudinal study in 107 transgender individuals, hormonal therapy reduced anxiety and depression symptoms but also functional impairments (Colizzi et al., 2014). This clearly shows a reduction in distress.

So how is the increasingly consistent finding that hormonal treatment is actually decreasing anxiety, depression, and suicides rates just an "additional data point" in the theory when it appears to account for much more of explaining how gender dysphoria can be treated than the new theory does? Likewise, regarding the statement of desisting in trans youth. What purpose or role does it play in the theory?

<u>Nonfactual, vague or inaccurate statements</u>

The abstract remains largely unexplained: "However, my theory uses a hierarchical executive function model to incorporate multiple reflexive factors (body ownership, gender typical/atypical behavior, and chronic distress) with the cognitive, reflective process of gender identity." Throughout the article the hierarchical executive function is not mentioned again or explained nor what is meant by reflexive factors (what exactly is reflexive in chronic distress?) and the cognitive reflective process of gender identity. Even the name of the theory is misleading: multisense theory, but it does not deal with multisenses. Chronic distress and the social behavior network are not directly related to senses. The author described the theory as "integrating information from multiple senses," but does not really mean senses here. So why this misleading term, which has a clear definition and is used in other fields of neuroscience? The author refers to the three concepts as reflexive senses, but does not explain why body ownership, gender typical/atypical behavior, and chronic distress are understood as reflexive senses. It is not explained why and how the sense of distress affects the sense of gender. For example, when does chronic distress lead to another mental disorder and when to gender dysphoria?

The social network also includes functions of mentalizing, empathy, social cognition, etc., functions that involve regions other than the ones mentioned. References here are rather old and do not incorporate newer findings.

What exactly is meant by "regions of the body-ownership network remained significant even after controlling for sexual orientation (Burke et al., 2017; Manzouri and Savic, 2019)"?

There is no "cellular activation measured by MEG."

The author tends to oversimplify and overstretch assumptions, such as: page 5: "two nodes of the social behavioral network (the network involved in gender-typical behavior)". There is no evidence for the social network being involved in gender-typical behavior.

It is also not clear what the relationship is between body ownership and incongruence between perceived gender identity and assigned gender. It seems the author often equates both terms.

"While the experimental evidence is strongest for the body-ownership/perception network," the experimental evidence for body ownership is not the strongest in transgender people. This theory explicitly targets only gender dysphoria but is used by the author to constantly refers to transgender.

This statement is also an oversimplification: “The multisense theory proposes that gender dysphoria is not merely due to static changes in anatomy, as in the previous opposite brain sex theory, but instead includes dynamic activity on interacting, functional networks”. There is existing evidence acknowledging that not only structural but also functional changes are present in gender dysphoria, so this is not at all new. Because this has been acknowledged and is known, it is not new as the author implies.

The hypothesis that partner preference is connected to the neurohormone vasopressin in brain regions related to social recognition should also deal with findings of environmental effects on vasopressin receptor expression in animal studies (e.g. Prounis et al., 2018).

Conclusion

It certainly is true that a “cause” for being transgender has not been found or identified. The author makes some interesting remarks that point in some directions that people have not gone into (as of yet) and that might be worthy of investigation. It would help fill in some of the gaps between the layers of neurobiology and reconciling hormonal, brain structural, and functional and behavioral data. Additionally, it would help elucidate other fields that have presently been understudied (e.g., link between hormones and sleep–wake cycle in trans people and the resultant effect of hormonal treatment). Likewise, a social behavioral network that is grounded in neurobiology has also not yet been examined and the question of minority stress on the individual and the individual’s biology also remains to be determined (especially including the different layers of neurobiology and neurochemistry). However, the paper is not substantial enough to warrant publication. The links drawn between at least two of the three networks are not compelling based on the small base of literature cited, especially since this literature is only of structural but not functional nature. Many of the present statements regarding functional implications are sheer conjecture. There is not enough evidence justifying this “new” theory and how it would actually advance the field.
